# Composition Analysis and Pharmacological Activity of Avocado/Soybean Unsaponifiable Products Used in the Treatment of Osteoarthritis

**DOI:** 10.3389/fphar.2021.781389

**Published:** 2022-01-10

**Authors:** Cécile Lambert, Gaëlle Bellemère, Gaëtan Boyer, Frank Ponelle, Thierry Bauer, Marie-Christine Legeny, Caroline Baudouin, Yves Henrotin

**Affiliations:** ^1^ MusculoSKeletal Innovative Research Lab, Center for Interdisciplinary Research on Medicines, University of Liège, Liège, Belgium; ^2^ Innovation, Research and Development, Laboratoires Expanscience, Epernon, France; ^3^ Department of Physical Therapy and Rehabilitation, Princess Paola Hospital, Vivalia, Marche-en-Famenne, Belgium; ^4^ Artialis S.A., Liège, Belgium

**Keywords:** unsaponifiable, avocado, soybean, osteoarthritis, chondrocytes

## Abstract

**Objective:** Avocado/soybean unsaponifiables (ASUs) are commonly used to treat OA symptoms. However, there are many ASU mixtures on the market with differing compositions and pharmacological activities. This study aimed to compare the composition and pharmacological activity of seven commercially available ASU products on human osteoarthritis chondrocytes.

**Methods:** The contents of the lipidic part of ASUs were characterized by gas chromatography analysis using a VARIAN 3400 chromatograph. The pharmacological activity of the ASU products was tested on human osteoarthritis chondrocytes cultured in alginate beads. Their effects were evaluated on aggrecan, interleukin (IL)-6 and -8, and matrix metalloproteases (MMP)-3 using immunoassays and on nitric oxide through measurement of nitrite *via* spectrometry.

**Results:** PIASCLEDINE-ExpASU^®^ showed a specific profile with the presence of chromatographic peaks corresponding to an alkyl furan fraction and alkyl triols. PIASCLEDINE-ExpASU^®^, Persemax, Insaponifiable 300, Arthrocen, and Arthocare contained quantifiable amounts of tocopherol, while tocopherol was undetectable in Avovida and Saponic. Squalene was found only in PIASCLEDINE-ExpASU^®^. The abundance of sterols varied depending on the product. PIASCLEDINE-ExpASU^®^ was the most active of the tested ASU products in inhibiting nitric oxide, IL-6, and IL-8 production by chondrocytes. With the exception of Saponic and Persemax, all the ASU mixtures either slightly or significantly increased aggrecan production. MMP-3 levels were significantly decreased by Insaponifiable 300 and PIASCLEDINE-ExpASU^®^ and significantly increased by Saponic.

**Conclusion:** The composition of PIASCLEDINE-ExpASU^®^ is different to that of the other evaluated ASU mixtures. This specific composition explains its better pharmacological activity, including the higher inhibitory effect on pro-inflammatory and pro-catabolic factors. Our findings are helpful in providing a basis for understanding the symptomatic effect of PIASCLEDINE-ExpASU^®^ in patients with osteoarthritis.

## Introduction

Osteoarthritis (OA) affects around 500 million people worldwide (Vos et al., 2017; Hunter et al., 2020) and is one of the most common causes of physical disability among older adults. Unfortunately, the chance of developing OA increases with age and is also associated with other common diseases such as diabetes (type 2) and cardiovascular diseases (Swain et al., 2020). The main hallmark of the disease is a progressive degradation of cartilage which is driven by a combination of mechanical and biochemical factors (Loeser et al., 2012). Chondrocytes play a key role by secreting locally abnormal quantities of catabolic and pro-inflammatory mediators. Therefore, regulation of chondrocyte metabolism remains a target for OA treatments, with a potential structure-modifying effect (Rahmati et al., 2016).

To date, there are few pharmaceutical options that can help to safely relieve symptoms and none that can cure OA (Glyn-Jones et al., 2015). Therefore, there is an important need for efficient treatments that can delay both the structural and clinical progression of the disease. One of the most widely used treatments for OA is a product composed of avocado and soybean unsaponifiables (ASUs), a member of the symptomatic slow-acting drugs in osteoarthritis (SYSADOA) family (Edgard Henrotin, 2018; Honvo et al., 2019). This product, called PIASCLEDINE-ExpASU^®^ is commercialized as a drug in many countries (PIASCLEDINE^®^300, Laboratoires Expanscience, Courbevoie, France). This is the only ASU product that has been rigorously investigated in robust randomized controlled trials (RCTs) (Maheu et al., 1998; Appelboom et al., 2001; Karel et al., 2010). It is a pharmaceutical-grade product composed of a specific ratio of avocado and soybean oil unsaponifiables (1:2 w/w) obtained by a patented process that influences the composition of the product (Maheu et al., 2014). Indeed, while the extraction of unsaponifiable fractions from soy oil does not represent any major hurdle, the extraction of unsaponifiables from the avocado pericarp is technically more complex because the high water content in the fruit tends to chemically modify a number of bioactive lipids *via* uncontrolled peroxidation and oxidization during the extraction process. To circumvent this, the process for extracting PIASCLEDINE-ExpASU^®^ has been adapted to include a drying step, whereby heat is used to remove the water from the avocado fruit before initiating ASU extraction (patent number: 10688142). It has been shown that a fraction of native furanic unsaponifiables specific to the avocado pericarp, identified as comprising persin compounds, undergoes cyclization when the fruit is submitted to heating (Kashman et al., 1969). This chemical transformation occurs during the PIASCLEDINE-ExpASU^®^ extraction process. The resulting persin-derived compounds represent a major unsaponifiable fraction in PIASCLEDINE-ExpASU^®^ that cannot be found in any other type of avocado extraction process, such as cold pressure and centrifugation, or in soy unsaponifiables (Farines et al., 1995). Similarly, the specific process used for preparation of PIASCLEDINE-ExpASU^®^ leads to enrichment of the avocado unsaponifiable fraction by alkyl triols, which has been previously described (Kashman et al., 1969; Néeman et al., 1970; Brown, 1973) (Laboratoires Expanscience, Internal data). The composition of PIASCLEDINE-ExpASU^®^ is complex and typically contains 35% sterols, 25% tocopherols, and 25% molecules from avocados that are obtained specifically for this process. The pharmacological activities of this compound have been well investigated, and the findings have been summarized in a narrative review study (Edgard Henrotin, 2018). In summary, PIASCLEDINE-ExpASU^®^ was found to stimulate aggrecan (AGG) and inhibit the production of interleukin (IL)-6 and -8, prostaglandin (PG) E2, and some matrix metalloproteases (MMPs) in chondrocytes. It also positively modulates the altered phenotype of OA subchondral bone osteoblasts and reduces the production of collagenases by synovial cells. At this time, four robust RCTs have all demonstrated a beneficial symptomatic effect of PIASCLEDINE-ExpASU^®^ in the treatment of hip or knee OA (Maheu et al., 1998; Appelboom et al., 2001; Karel et al., 2010; Anon, 2021a).

However, in the past years, many food supplements based on ASUs that have emerged on the market in recent years are claiming to have similar pharmacological activities and alleging more or less clearly the same clinical and safety profile as PIASCLEDINE-ExpASU^®^ (Ghasemian et al., 2016).

This study aimed to compare the composition and pharmacological activity of products composed of ASUs available on the market, including PIASCLEDINE-ExpASU^®^. These products were chosen because they were the most representative products in different regions. More precisely, we studied the chromatographic pattern of these compounds and their effects on human OA chondrocytes in alginate bead cultures.

## Materials and Methods

### Products Investigated

The following products were included in the study: Arthocare (Bonapharm S.A.C., Lima, Peru), Arthrocen (Pharmin United States, LLC, San Jose, CA, United States), Avovida (Pharma Nature, Saint Hippolyte du Fort, France), Insaponifiable 300 (GIPHAR Group, Paris, France), Persemax (Laboratorios Synthesis SAS, Bogota, Colombia), PIASCLEDINE-ExpASU^®^ (Laboratoires Expanscience, Courbevoie, France), and Saponic (Laboratorio Gador La Paz, Montevideo, Uruguay). All products were presented as 300 mg capsules.

### Analytical Assays

For each product, the mass of 20 individual capsules was weighed and recorded. The content of the capsules was collected and subjected to Soxhlet extraction using chloroform. Soxhlet extraction is a very standard and well-recognized method that leads to the extraction of water-insoluble and slightly water-soluble organics identified as semi-volatile organic compounds (SVOCs) (Abubakar and Haque, 2020). The mass of the SVOC fraction was determined by gravimetry and recorded as the lipidic part. The contents of the lipidic part were characterized by gas chromatography analysis using a VARIAN 3400 chromatograph equipped with a septum-equipped programmable injector (SPI), a flame ionization detector (FID), and a capillary column with 5%-phenyl-95%-methylpolysiloxane as the stationary phase and helium as the mobile phase. A solution of squalene at 0.2% (m/v) in hexane and a solution of avocado oil unsaponifiables at 0.4% (m/v) and soybean oil unsaponifiables at 0.8% (m/V) in chloroform were used as standards for the identification of unsaponifiables. Quantification was performed by gas chromatography using purified alkyl furans and alkyl triols in addition to aliphatic alkane fractions from avocado, purified sterol fractions from soy, and tocopherol standards.

### Patients

OA human articular cartilage was obtained from 12 different patients (five women and seven men; mean age of 62 ± 6.2 years) with knee OA at the time of total knee joint replacement surgery. All the subjects provided their informed consent, and ethical approval (ethics committee agreement of Liège University, no. B70720108313) was granted for this study.

### Cartilage Processing and Chondrocyte Culture in Alginate Beads

To allow testing the product on primary human OA chondrocytes, which are one of the therapeutic targets of ASU, we used freshly isolated chondrocytes expressing inflammatory phenotypes defined by a high rate of IL-6 production (up to 40 ng/µg DNA). To obtain OA chondrocytes, the following process was used: 1. On dissection, the femoral, patellar, and tibial articular surfaces were evaluated for gross morphological modifications of cartilage according to the Collins diagram (Collins D and McElligott T, 1960). The severity of lesions was, thus, recorded for each sample. Different grades were considered: 0, normal white cartilage in all areas examined; I, the presence of a yellow–gray area with some superficial fibrillations on one or more articular surfaces; II, irregular surface with deep fibrillations on one or more articular surfaces; III, subchondral bone denudation on one or more articular surfaces, in less than 50% of the most damage compartment; and IV, subchondral bone denudation on more than 50% of the articular surface at least in one tibial plateau. All donors showed OA cartilage lesions of grade III to IV. 2. We evaluated the IL-6 levels produced by cultured chondrocytes using an immunoassay.

Full-depth articular cartilage was excised and immersed in Dulbecco’s modified Eagle’s medium (DMEM) (with phenol red and 4.5 g/L of glucose) supplemented with N-(2-hydroxyethyl)piperazine-N’-(2-ethanesulfonic acid) (HEPES) 10 mM, penicillin (100 U/ml), and streptomycin (0.1 mg/ml) (all from Biowest, Nuaillé, France). After three washes, the chondrocytes were released from the cartilage by sequential enzymatic digestions with 0.5 mg/ml of hyaluronidase type IV-S (Sigma-Aldrich, Bornem, Belgium) for 30 min at 37°C, 1 mg/ml of pronase E (Merck, Leuven, Belgium) for 1 h at 37°C, and 0.5 mg/ml clostridial collagenase IA (Sigma-Aldrich) for 16–20 h at 37°C. The enzymatically isolated cells were then suspended in alginate beads, as previously described (Sanchez et al., 2002), and maintained in culture for 12 days in DMEM supplemented with 1% ITS+ (Lonza, Verviers, Belgium), 10 mM HEPES, penicillin (100 U/ml) and streptomycin (0.1 mg/ml), 200 µg/ml of glutamine (Lonza), 50 µg/ml of ascorbic acid (Sigma-Aldrich), and 2 mM proline (Sigma-Aldrich). The cells remained in this culture medium (washout medium) for 48 h. After this washout period, the culture medium was changed every 3 days, and the collected supernatants were stored at −20°C until analysis. The chondrocytes were cultured for 12 days either without any ASU (negative control, Ctl) or with 10 µg/ml PIASCLEDINE-ExpASU^®^ or one of the six other nutraceutical products being evaluated in comparison, namely, Arthocare, Avovida, Arthrocen, Insaponifiable 300, Persemax, or Saponic (3 wells/condition). At the end of the culture period, the culture medium was carefully discarded, and the beads were dissolved in 1 ml of 0.1 M citrate for 10 min. The resulting suspension was centrifuged at 1,200 rpm for 10 min. With this method, two fractions were collected: the supernatant containing macromolecules originating from the further-removed matrix (FRM) and a pellet containing cells with their associated matrix (CM).

Twelve cultures have been performed. Each culture was performed with chondrocytes coming from one single cartilage specimen. Given the limited number of cells per specimen, it was not possible to test all products on the same specimen. Therefore, the products were separated into two groups. Each group was composed of 3 products and the reference product PIASCLEDINE-ExpASU^®^. Each product group has been tested on six independent cultures. Each culture condition was performed in triplicates.

### DNA Assay

The DNA content of the culture was measured according to the fluorometric method using Hoechst (Labarca and Paigen, 1980).

### Immunoassay for Aggrecan, Interleukin-6 and -8, and MMP-3

The total AGG production corresponded to the AGG accumulated in the alginate beads. AGG at D12 and IL-6, IL-8, and MMP-3 only in the supernatant (cumulative production D3_D6_D9 and D12) were measured by specific enzyme-amplified sensitivity immunoassays (Invitrogen, Merelbeke, Belgium).

### LDH Assay

Cell viability was estimated by quantifying the release of LDH in the culture supernatant, as previously described (Mathy-Hartert et al., 2009). A sample of the supernatant or dilutions of the standard solution (LDH from rabbit muscle) was mixed with Tris buffer [10 mM Tris-HCl (pH 8.5) and, 0.1% bovine serum albumin] containing 800 mM lactate. Then, a colorimetric reagent containing 1.6 mg/ml iodonitrotetrazolium chloride (Sigma-Aldrich), 4 mg/ml nicotinamide adenine dinucleotide (Roche Diagnostics, Brussels, Belgium), and 0.4 mg/ml phenazine methosulfate (Sigma-Aldrich) was added, and the absorbance at 492 nm was read after 10 min of incubation at room temperature.

### Nitric Oxide Assay

Nitrite and nitrate are stable end products of nitric oxide. Nitrate was reduced to nitrite by its addition to the supernatant of nitrate reductase (0.25 U/ml) for 20 min at 37°C. The cumulative nitrite concentrations in conditioned culture supernatants after 3, 6, 9, and 12 days of culture were determined *via* a spectrophotometric method based upon the Griess reaction. The absorption was measured at 540 nm. Sodium nitrite (NaNO_2_) was used for calibration.

### Statistical Analysis

Data from *in vitro* experiments were analyzed and compared using a paired Student’s *t*-test and bilateral hypothesis followed by Bonferroni adjustment. Differences were considered significant when *p* < 0.05. Data are presented as histograms. The exact *p* values are provided, and asterisk representations were also performed. All data were analyzed by R software.

## Results

### ASU Products Composition

For all the products except Piascledine^®^300, the lipidic part extracted by the Soxhlet device represented less than 300 mg/capsule lipidic part, while the PIASCLEDINE-ExpASU^®^ capsules contained 305.5 mg of the lipidic part (Table 1).

**TABLE 1:** Product composition by comparison of gas chromatography profiles. The results are expressed in milligrams per capsule (mg/capsule) for each indicated fraction.

	Piascledine E-ASU	Persemax	Saponic	Arthrocen	Arthocare	Avovida	Insaponifiable 300
Mass (mg/capsule)	307.0	356.6	352.0	372.5	773.3	296.8	381.0
Lipid content (mg/capsule)	305.5	185.1	133.0	212.0	666.8	220.8	201.4
Alkyl furans (mg/capsule)	65.2 ± 2.0	Absence	Absence	Absence	Absence	Absence	Absence
Alkyl triols & aliphatic alkanes (mg/capsule)	3.0–15.0	Absence	Absence	Absence	Absence	Absence	Absence
Tocopherols (mg/capsule)	73.7 ± 4.1	1.8	Absence	1.5	0.1	Absence	1.8
Sterols (mg/capsule)	106.0 ± 5.1	124.0	102.0	126.8	195.4	138.4	154.2
Squalene (mg/capsule)	2.0–30.0	Absence	Absence	Traces	Traces (0.2)	Absence	Traces (0.4)

**FIGURE 1:**
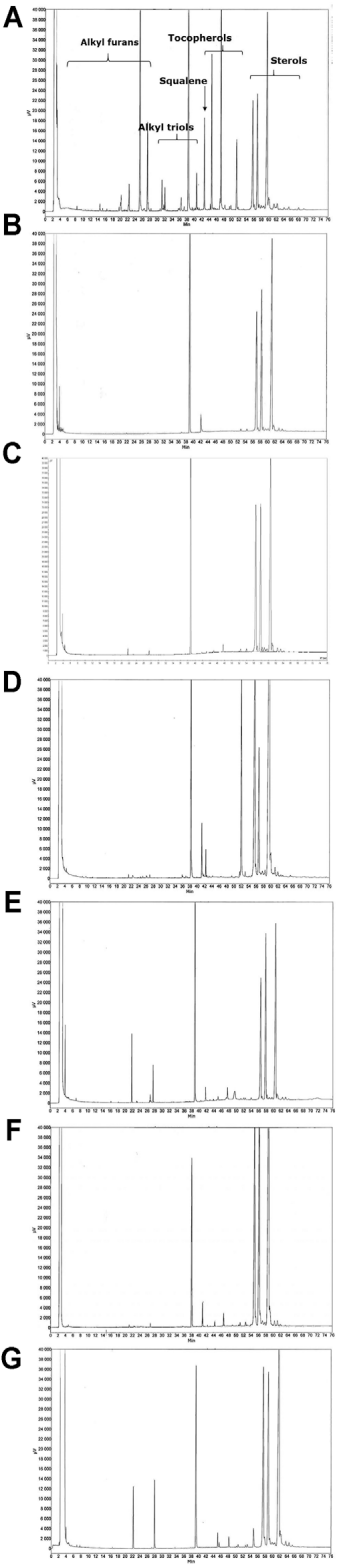
Typical gas chromatography profiles of the products. **(A)** PIASCLEDINE-ExpASU^®^; **(B)** Saponic; **(C)** Persemax; **(D)** Arthocare; **(E)** Avovida; **(F)** Insaponifiable 300; and **(G)** Arthrocen. For each product, the mass of 20 individual capsules was weighed and recorded. The content of the capsules was collected and subjected to a Soxhlet extraction using chloroform. The mass of the SVOC fraction was determined by gravimetry and recorded as the lipidic part. The contents of the lipidic part were characterized by gas chromatography analysis using a VARIAN 3400 chromatograph. The composition of the products was compared by superimposing their chromatographic profiles. The purified standard of unsaponifiable fractions from avocado and soybean was used for characterization and quantification.

The composition of the products was compared by superimposing their chromatographic profiles (Figure 1). The purified standard of unsaponifiable fractions from avocados and soybeans was used for characterization and quantification. PIASCLEDINE-ExpASU^®^ showed a specific profile with the presence of chromatographic peaks corresponding to alkyl furans and alkyl triols. Subsequent quantification showed that the PIASCLEDINE-ExpASU^®^ product contained 65.2 ± 2.0 mg/capsule of alkyl furans and 3.0–15.0 mg/capsule of alkyl triols and aliphatic alkane fractions. All other products investigated in our study were devoid of these unsaponifiable compounds (Table 1).

Arthocare (0.1 mg/capsule), Arthrocen (1.5 mg/capsule), Insaponifiable 300 (1.8 mg/capsule), Persemax (1.8 mg/capsule), and PIASCLEDINE-ExpASU^®^ (73.7 mg/capsule) contained quantifiable amounts of tocopherol, while tocopherol was undetectable in Avovida and Saponic. Only PIASCLEDINE-ExpASU^®^ contained squalene (2 mg/capsule). The sterol content varied depending on the product: 102 mg/capsule in Saponic, 106 mg/capsule in PIASCLEDINE-ExpASU^®^, 124 mg/capsule in Persemax, 126.8 mg/capsule in Arthrocen, 138.4 mg/capsule in Avovida, 154.2 mg/capsule in Insaponifiable 300, and 195.4 mg/capsule in Arthocare.

### NO Production

Primary OA human chondrocytes were cultured for 12 days in alginate beads with or without ASU products. Cell viability, evaluated by LDH release and DNA content, were not affected by the tested compounds (data not shown).

**FIGURE 2:**
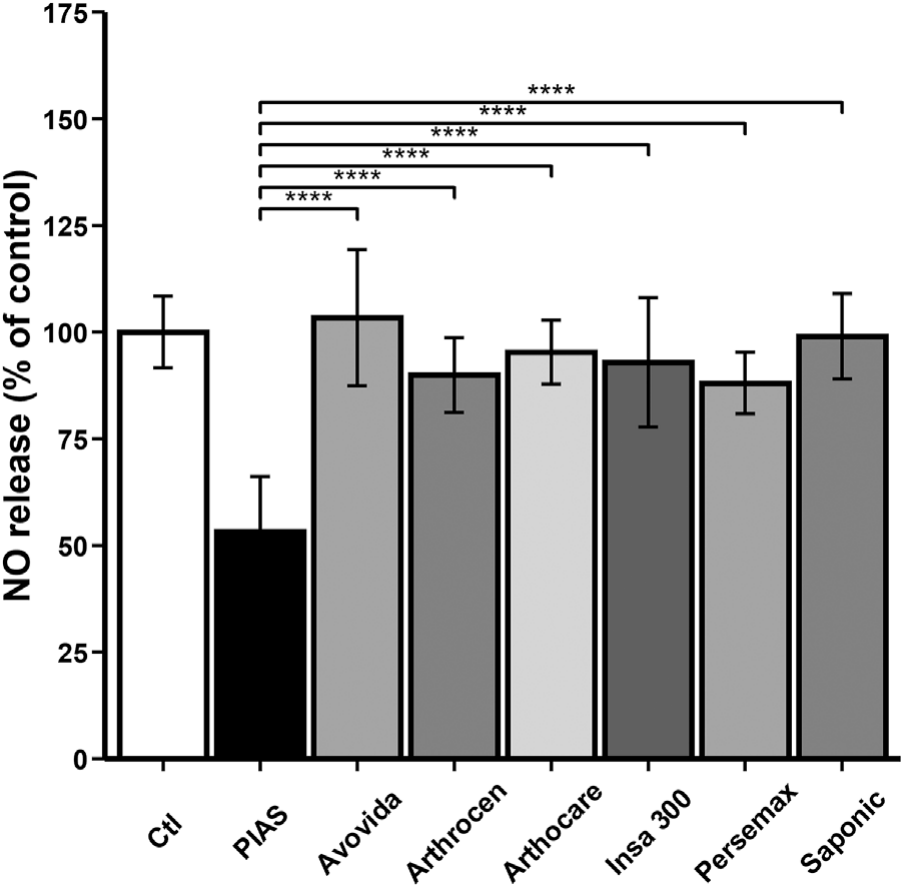
Effect of ASU products tested on total NO production. The chondrocytes were cultured for 12 days either without any ASU (negative control, Ctl) or with 10 µg/ml of either PIASCLEDINE-ExpASU^®^ (PIAS) or one of the six other nutraceutical products being evaluated in comparison, namely, Avovida, Arthrocen, Arthocare, Insaponifiable 300 (Insa 300), Persemax, or Saponic. Each product has been tested on six independent cultures. Each culture condition was performed in triplicates. The results are expressed as the percent control and represented by the mean ± SD. ****p < 0.0001 versus PIASCLEDINE-ExpASU^®^.

Under basal conditions, the NO production was 69.11 ± 20.08 nmoL/µg of DNA. As illustrated in Figure 2, Arthrocen, PIASCLEDINE-ExpASU^®^, and Persemax significantly decreased NO production (Ctl vs. PIASCLEDINE-ExpASU^®^: *p* < 0.0001; Ctl vs. Arthrocen: *p* = 0.01563; Ctl vs. Persemax: *p* = 0.00044). However, the effects of PIASCLEDINE-ExpASU^®^ on NO production were significantly higher than those of other compounds (*p* < 0.0001). In contrast, Arthrocare, Avovida, Insaponifiable 300, and Saponic had no significant effect on NO production.

### AGG Production

**FIGURE 3:**
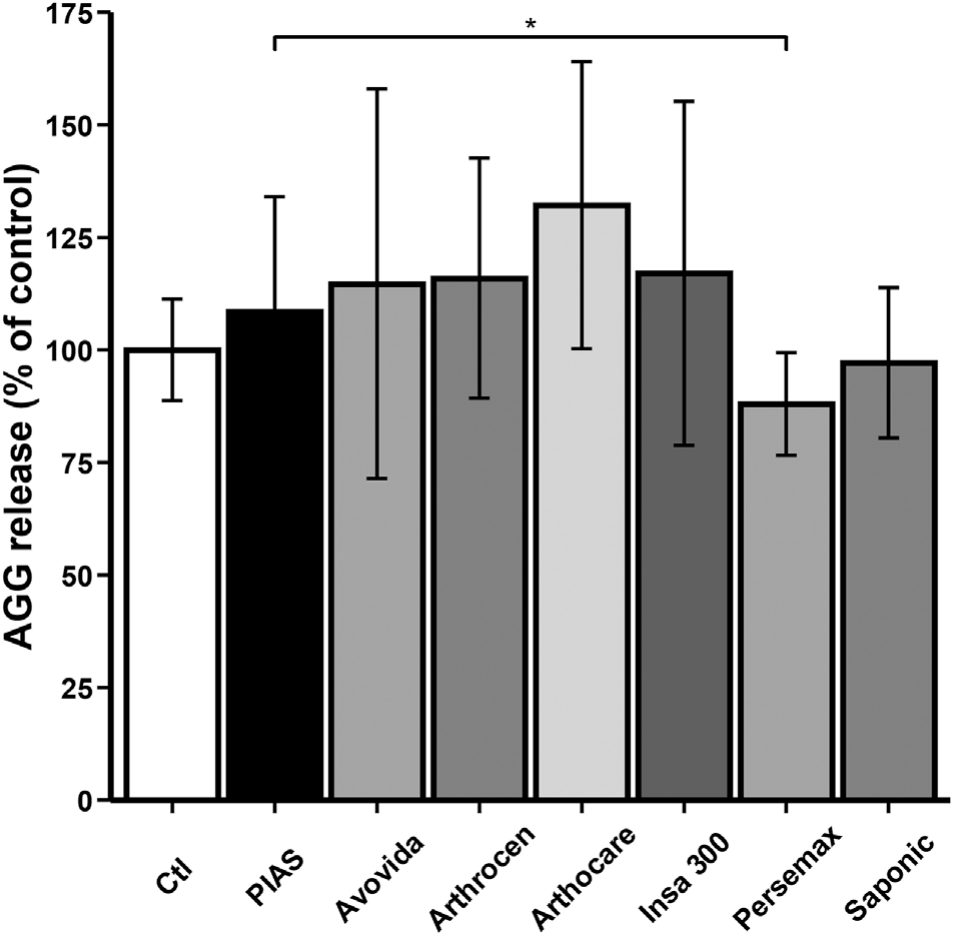
Effect of ASU products tested on total AGG production. The chondrocytes were cultured for 12 days either without any ASU (negative control, Ctl) or with 10 µg/ml of either PIASCLEDINE-ExpASU^®^ (PIAS) or one of the six other nutraceutical products being evaluated in comparison, namely, Avovida, Arthrocen, Arthocare, Insaponifiable 300 (Insa 300), Persemax, or Saponic. Each product has been tested on six independent cultures. Each culture condition was performed in triplicates. The results are expressed as the percent control and represented by the mean ± SD. *p < 0.05 versus PIASCLEDINE-ExpASU^®^.

Except for Persemax and Saponic, all other products either slightly or significantly increased the total AGG production by human OA chondrocytes (Ctl vs. PIASCLEDINE-ExpASU^®^: *p* = 0.10116; Ctl vs. Arthrocen: *p* = 0.04979; Ctl vs. Arthocare: *p* = 0.00010; Ctl vs. Persemax: *p* = 0.00063) (Figure 3).

### Interleukin-6

**FIGURE 4:**
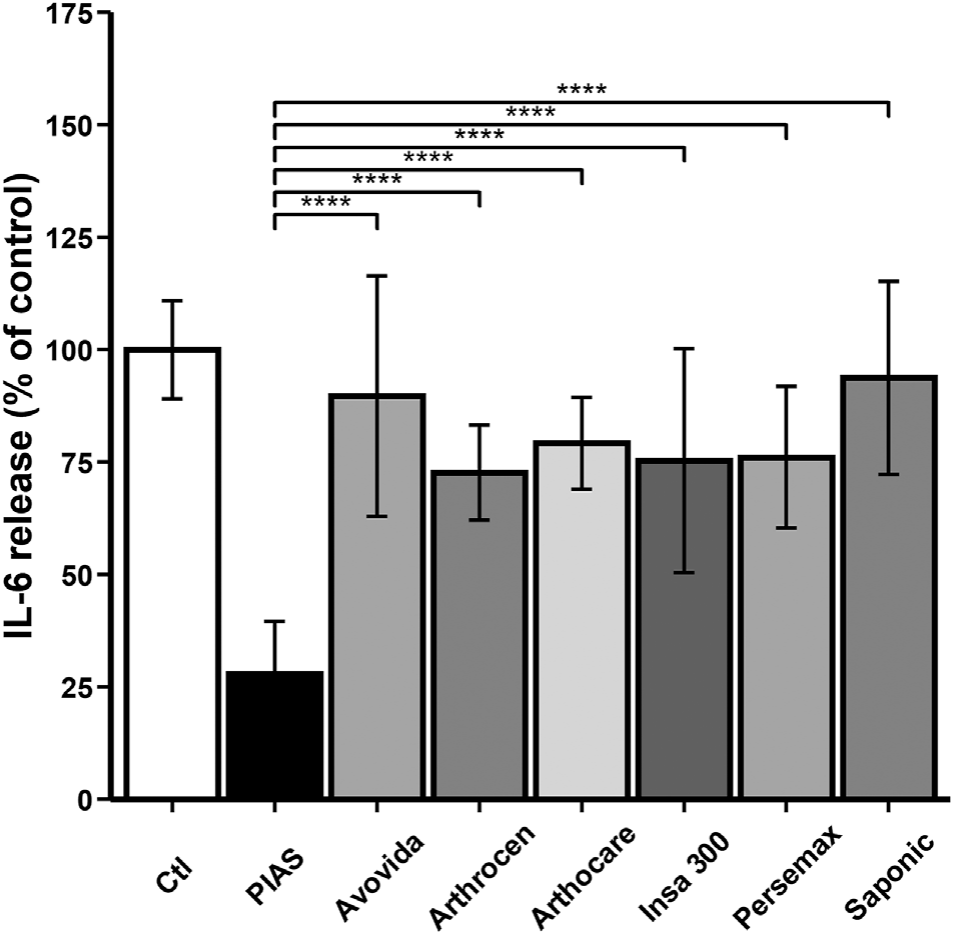
Effect of ASU products tested on total IL-6 production. The chondrocytes were cultured for 12 days either without any ASU (negative control, Ctl) or with 10 µg/ml of either PIASCLEDINE-ExpASU^®^ (PIAS) or one of the six other nutraceutical products being evaluated in comparison, namely, Avovida, Arthrocen, Arthocare, Insaponifiable 300 (Insa 300), Persemax, or Saponic. Each product has been tested on six independent cultures. Each culture condition was performed in triplicates. The results are expressed as the percent control and represented by the mean ± SD. ****p < 0.0001 versus PIASCLEDINE-ExpASU^®^.

Except for Avovida and Saponic, all other products significantly decreased IL-6 production by human OA chondrocytes (Ctl vs. PIASCLEDINE-ExpASU^®^: *p* < 0.0001; Ctl vs. Arthrocen: *p* = 0.00031; Ctl vs. Arthocare: *p* = 0.00244; Ctl vs. Insaponifiable 300: *p* = 0.00038; Ctl vs. Persemax: *p* = 0.00014) (Figure 4). However, the inhibitory effect of PIASCLEDINE-ExpASU^®^ was significantly higher than that of the other compounds (*p* < 0.0001).

### Interleukin-8

**FIGURE 5:**
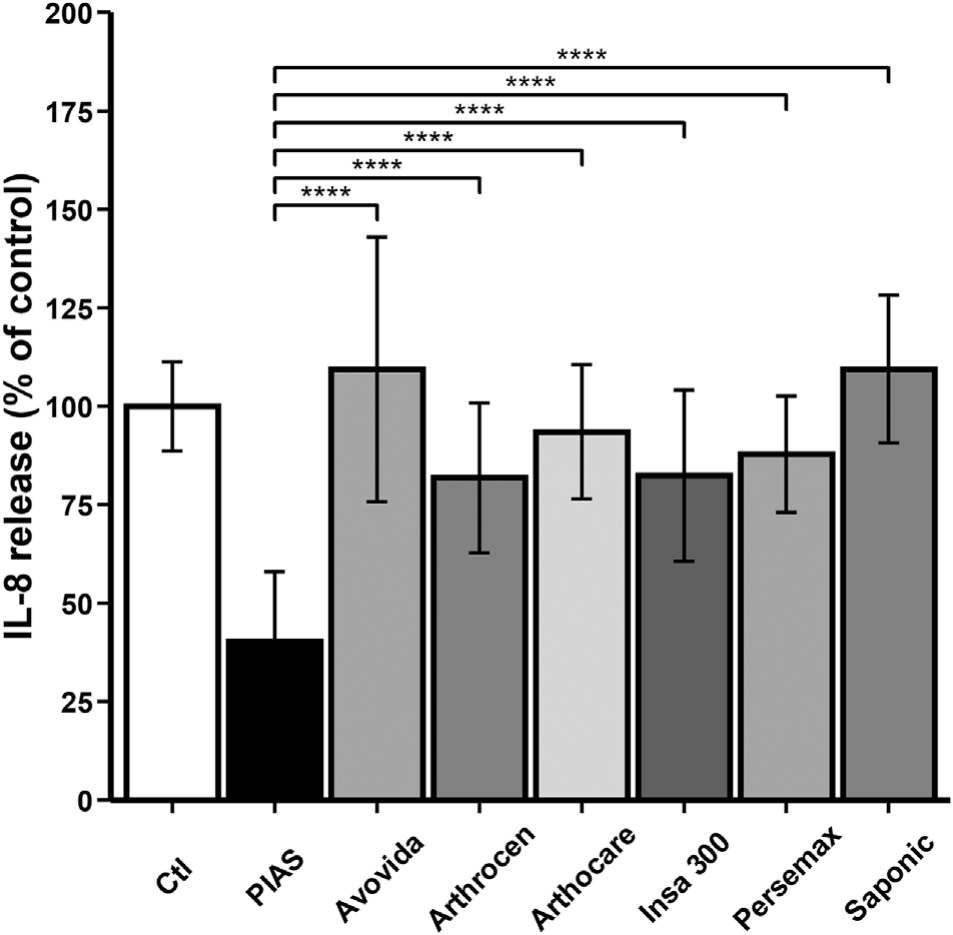
Effect of ASU products tested on total IL-8 production. The chondrocytes were cultured for 12 days either without any ASU (negative control, Ctl) or with 10 µg/ml of either PIASCLEDINE-ExpASU^®^ (PIAS) or one of the six other nutraceutical products being evaluated in comparison, namely, Avovida, Arthrocen, Arthocare, Insaponifiable 300 (Insa 300), Persemax, or Saponic. Each product has been tested on six independent cultures. Each culture condition was performed in triplicates. The results are expressed as the percent control and represented by the mean ± SD. ****p < 0.0001 versus PIASCLEDINE-ExpASU^®^.

As illustrated in Figure 5, except for Avovida and Arthocare, all other ASU products significantly decreased IL-8 production by chondrocytes (Ctl vs. PIASCLEDINE-ExpASU^®^: *p* < 0.0001; Ctl vs. Arthrocen: *p* = 0.00711; Ctl vs. Insaponifiable 300: *p* = 0.00658; Ctl vs. Persemax: *p* = 0.00192; Ctl vs. Saponic: *p* = 0.00603). However, the inhibitory effect of PIASCLEDINE-ExpASU^®^ was significantly higher than that of the other compounds (*p* < 0.0001).

### Matrix Metalloprotease-3

**FIGURE 6:**
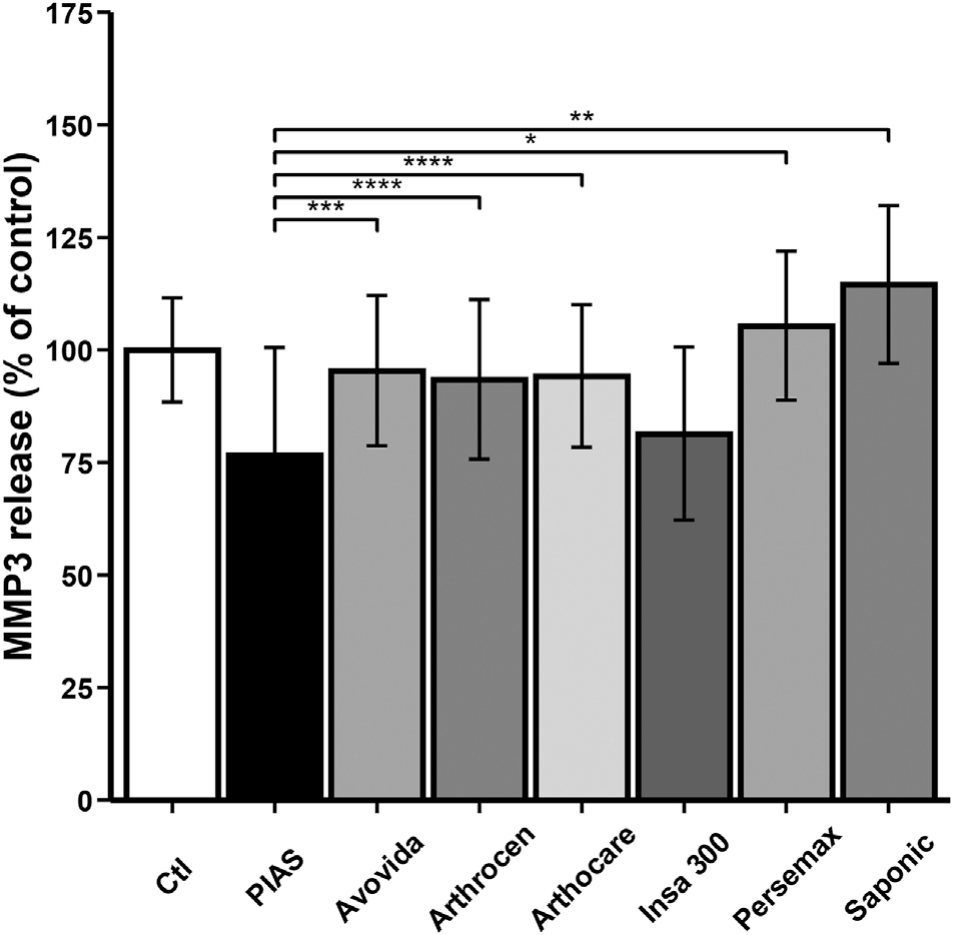
Effect of ASU products tested on total MMP-3 production. The chondrocytes were cultured for 12 days either without any ASU (negative control, Ctl) or with 10 µg/ml of either PIASCLEDINE-ExpASU^®^ (PIAS) or one of the six other nutraceutical products being evaluated in comparison, namely, Avovida, Arthrocen, Arthocare, Insaponifiable 300 (Insa 300), Persemax, or Saponic. Each product has been tested on six independent cultures. Each culture condition was performed in triplicates. The results are expressed as the percent control and represented by the mean ± SD. *p < 0.05 versus PIASCLEDINE-ExpASU^®^, **p < 0.01 versus PIASCLEDINE-ExpASU^®^, ***p < 0.001 versus PIASCLEDINE-ExpASU^®^, and ****p < 0.0001 versus PIASCLEDINE-ExpASU^®^.

PIASCLEDINE-ExpASU^®^ and Insaponifiable 300 significantly decreased the basal production of MMP-3 (Ctl vs. PIASCLEDINE-ExpASU^®^: *p* = 0.00019; Ctl vs. Insaponifiable 300: *p* = 0.00204) while Arthocare, Arthrocen, Avovida, and Persemax had no significant effects, and Saponic significantly increased MMP-3 production (Ctl vs. Saponic: *p* = 0.04103) (Figure 6).

## Discussion

ASU products are commonly used to treat OA patients. They are members of a class of drugs called SYSADOA, which have been recommended by many scientific and medical societies for relieving OA symptoms (Jordan et al., 2003; Zhang et al., 2005; Anon 2021b). However, ASU products may differ in their composition as a consequence of their manufacturing process. Until now, the composition of the main ASU products available on the market had never been compared. Moreover, it has never been demonstrated how the composition of ASU products might influence their pharmacological activity. For the first time, our study compared the chromatographic profiles of seven ASU products commercially available with the aim of identifying differences in their pharmacological activity on human OA chondrocytes. As chondrocytes are key players in cartilage degradation in OA, our study also contributes to identifying which ASU product components may be significant in explaining differences in the pharmacological activity of ASU products.

Our characterization clearly of ASU products shows that PIASCLEDINE-ExpASU^®^ has a unique composition characterized by the presence of alkyl furans, alkyl triols, and squalene. These compounds were absent in all of the other mixtures, indicating that these molecules could be responsible for the different pharmacological activity profiles of PIASCLEDINE-ExpASU^®^. Indeed, PIASCLEDINE-ExpASU^®^ was the most effective inhibitor of NO, IL-6, IL-8, and MMP-3. The unique formulation of PIASCLEDINE-ExpASU^®^ is obtained by a particular proprietary-patented process. Our results confirmed those of a previous study conducted by the manufacturer (Msika et al., 2008), which demonstrated that the commercial nutraceutical products called Dasuquin and Avoca ASU (Nutramax, United States) totally differ in composition from the common and natural sterol-based avocado and soybean unsaponifiables and also from Piascledine^®^300, namely, in the absence of or not having detectable levels of specific molecules from common natural avocado unsaponifiables such as the key molecule citrostadienol, the absence of alkyl furans, alkyl triols, and squalene and finally, the presence of brassicasterol, which is highly specific for rapeseed oil and related unsaponifiables (Msika et al., 2008).

Another major constituting element of ASU mixtures are the phytosterols beta-sitosterol, campesterol, and stigmasterol. Lippiello et al. compared the sterol composition of NMX-1000™ and Piasceldine^®^300 and tested the influence of the sterol content on the upregulation of glycosaminoglycan and collagen synthesis by bovine chondrocytes *in vitro* and on the upregulation of PGE_2_ in an IL-1–induced *in vitro* model of articular cartilage breakdown (Lippiello et al., 2008). They reported that PIASCLEDINE-ExpASU^®^ has a different gas chromatographic profile to NMX-1000™ with three additional major unidentified peaks, while the total content of sterols was similar. This confirms our data showing that PIASCLEDINE-ExpASU^®^ has a unique composition. However, in contrast to our study, they showed that both ASU products, equalized to contain equivalent sterol contents, had similar metabolic effects on articular chondrocytes. The two products inhibited prostaglandin synthesis, metalloprotease activity, and the release of radiolabeled sulfate from pre-labeled cartilage. This discrepancy can be explained by differences in the methodological approach used to test the pharmacological activity of ASUs. We used human chondrocytes in alginate beads, while Lippiello et al. used bovine monolayer chondrocytes and cartilage explants. Furthermore, the length of time of cell exposure to the tested components also drastically differed. In our study, cells were treated for 12 days while in the study of Lippiello et al., the exposure time was shorter. Another major difference between the two studies was that we used doses of ASUs by weight in contrast to Lippiello et al. who standardized according to the sterol content. Finally, the investigated parameters were different. We tested the effects of ASUs on NO, IL-6, and IL-8, main inflammatory mediators implicated in OA pathogenesis. Through cytokine-induced MAP kinases, NF-κB activation, and oxidative phosphorylation, these mediators contribute to systemic inflammation (Lambert et al., 2021). These parameters were not explored by Lippiello et al. Interestingly, in our study, this product did not have the highest level of sterols of the evaluated ASUs but was the most active. Indeed, compared to PIASCLEDINE-ExpASU^®^, Arthocare contained almost twice the amount ofsterols but did not demonstrate effective inhibition of MMP-3, NO, and IL-8 and was significantly less effective on IL-6 than PIASCLEDINE-ExpASU^®^. Furthermore, surprisingly, there was an absence of effects for Avovida. This can be explained by the absence of four key components in its composition. In addition to the absence of alkyl furans, alkyl triols, and squalene, Avovida does not contain tocopherols. These data suggest that tocopherols could play a role in determining the pharmacological activities of ASU mixtures.

There are some limitations to our study with regard to interpreting the impact of composition on the pharmacological activity. One is the lack of definitive identification of the various minor components of the ASUs. Another is the small number of parameters investigated. It is possible that when assessing other parameters, the activity of ASU products would show different trends. Finally, we investigated a limited number of products. We cannot exclude the possibility that other commercially available products not studied here may show effects comparable to PIASCLEDINE-ExpASU^®^.

In conclusion, our study demonstrates that PIASCLEDINE-ExpASU^®^ has a unique composition characterized by the presence of alkyl furans, alkyl triols, and squalene. This specific composition can explain its higher efficacy on pro-inflammatory and pro-catabolic mediators compared to other ASU products. Our data also confirm that PIASCLEDINE-ExpASU^®^ shows beneficial effects on the chondrocyte metabolism *via* AGG, which could explain, in part, its clinical efficacy in OA patients. Therefore, to extrapolate the clinical data obtained with one ASU formulation to another one remains fully speculative. Our findings justify investigating each ASU product in clinical trials before claiming any efficacy with respect to OA symptoms.

## Data Availability

The raw data supporting the conclusions of this article will be made available by the authors, without undue reservation.

## Abbreviations

AGG, aggrecan; ASU, avocado and soybean unsaponifiables; CM, cells with their associated matrix; DMEM, Dulbecco’s modified Eagle’s medium; DNA, deoxyribonucleic acid; FID, flame ionization detector; FRM, further-removed matrix; HEPES, N-(2-hydroxyethyl)piperazine-N’-(2-ethanesulfonic acid); IL-, interleukin; LDH, lactate dehydrogenase; MMP, matrix metalloproteases; NaNO_2_, sodium nitrite; NO, nitric oxide; OA, osteoarthritis; PG, prostaglandin; RCT, randomized controlled trials; SPI, septum-equipped programmable injector; SVOC, semi-volatile organic compounds; SYSADOA, symptomatic slow-acting drugs in osteoarthritis.
